# Multicountry Distribution and Characterization of Extended-spectrum β-Lactamase–associated Gram-negative Bacteria From Bloodstream Infections in Sub-Saharan Africa

**DOI:** 10.1093/cid/ciz450

**Published:** 2019-10-30

**Authors:** Trevor Toy, Gi Deok Pak, Trung Pham Duc, James I Campbell, Muna Ahmed El Tayeb, Vera Von Kalckreuth, Justin Im, Ursula Panzner, Ligia Maria Cruz Espinoza, Daniel Eibach, Denise Myriam Dekker, Se Eun Park, Hyon Jin Jeon, Frank Konings, Ondari D Mogeni, Leonard Cosmas, Morten Bjerregaard-Andersen, Nagla Gasmelseed, Julian T Hertz, Anna Jaeger, Ralf Krumkamp, Benedikt Ley, Kamala Thriemer, Leon Parfait Kabore, Aissatou Niang, Tiana Mirana Raminosoa, Emmanuel Sampo, Nimako Sarpong, Abdramane Soura, Ellis Owusu-Dabo, Mekonnen Teferi, Biruk Yeshitela, Sven Poppert, Jürgen May, Jerome H Kim, Yun Chon, Jin Kyung Park, Abroaham Aseffa, Robert F Breiman, Heidi Schütt-Gerowitt, Peter Aaby, Yaw Adu-Sarkodie, John A Crump, Raphaël Rakotozandrindrainy, Christian G Meyer, Amy Gassama Sow, John D Clemens, Thomas F Wierzba, Stephen Baker, Florian Marks

**Affiliations:** 1 International Vaccine Institute, Seoul, South Korea; 2 Oxford University Clinical Research Unit, Ho Chi Minh City, Vietnam; 3 Faculty of Medicine, University of Gezira, Wad Medani, Sudan; 4 Bernhard Nocht Institute for Tropical Medicine, Hamburg, Germany; 5 German Center for Infection Research, Hamburg-Borstel-Lübeck, Germany; 6 Department of Medicine, Cambridge University, United Kingdom; 7 Kenya Medical Research Institute-Centre for Global Health Research (KEMRI-CGHR), Nairobi; 8 Centers for Disease Control and Prevention, KEMRI Complex, Nairobi, Kenya; 9 Bandim Health Project, Bissau, Guinea-Bissau; 10 Research Center for Vitamins and Vaccines, Copenhagen, Denmark; 11 Faculty of Science, University of Hafr Al Batin, Saudi Arabia; 12 Kilimanjaro Christian Medical Centre, Moshi, Tanzania; 13 Division of Infectious Diseases and International Health, Duke University Medical Center, Durham, North Carolina; 14 Global and Tropical Health Division, Menzies School of Health Research, Charles Darwin University, Australia; 15 Schiphra Hospital, Ouagadougou, Burkina Faso; 16 Institute Pasteur de Dakar, Senegal; 17 University of Antananarivo, Antananarivo, Madagascar; 18 Institut Supérieur des Sciences de la Population, University of Ouagadougou, Burkina Faso; 19 Kumasi Centre for Collaborative Research in Tropical Medicine, Kwame Nkrumah University of Science and Technology (KNUST), Ghana; 20 Department of Global and International Health, School of Public Health, KNUST, Kumasi, Ghana; 21 Armauer Hansen Research Institute, Addis Ababa, Ethiopia; 22 Infectious Diseases Department, University Hospital Eppendorf, Hamburg, Germany; 23 Global Health Institute, Emory University, Atlanta, Georgia; 24 Institute of Medical Microbiology, University of Cologne, Germany; 25 Kwame Nkrumah University of Science and Technology, Kumasi, Ghana; 26 Duke Global Health Institute, Duke University, Durham, North Carolina; 27 Centre for International Health, University of Otago, Dunedin, New Zealand; 28 Institute of Tropical Medicine, Eberhard-Karls University Tübingen, Germany; 29 Duy Tan University, Da Nang, Vietnam; 30 Université Cheikh Anta Diop de Dakar, Senegal; 31 International Centre for Diarrheal Disease Research, Dhaka, Bangladesh; 32 University of California, Fielding School of Public Health, Los Angeles; 33 School of Medicine, Korea University, Seoul, South Korea

**Keywords:** extended-spectrum β-lactamase, ESBL, antibiotic resistance, antimicrobial resistance, Africa, surveillance

## Abstract

**Background:**

Antimicrobial resistance (AMR) is a major global health concern, yet, there are noticeable gaps in AMR surveillance data in regions such as sub-Saharan Africa. We aimed to measure the prevalence of extended-spectrum β-lactamase (ESBL) producing Gram-negative bacteria in bloodstream infections from 12 sentinel sites in sub-Saharan Africa.

**Methods:**

Data were generated during the Typhoid Fever Surveillance in Africa Program (TSAP), in which standardized blood cultures were performed on febrile patients attending 12 health facilities in 9 sub-Saharan African countries between 2010 and 2014. Pathogenic bloodstream isolates were identified at the sites and then subsequently confirmed at a central reference laboratory. Antimicrobial susceptibility testing, detection of ESBL production, and conventional multiplex polymerase chain reaction (PCR) testing for genes encoding for β-lactamase were performed on all pathogens.

**Results:**

Five hundred and five pathogenic Gram-negative bloodstream isolates were isolated during the study period and available for further characterization. This included 423 Enterobacteriaceae. Phenotypically, 61 (12.1%) isolates exhibited ESBL activity, and genotypically, 47 (9.3%) yielded a PCR amplicon for at least one of the screened ESBL genes. Among specific Gram-negative isolates, 40 (45.5%) of 88 *Klebsiella* spp., 7 (5.7%) of 122 *Escherichia coli*, 6 (16.2%) of 37 *Acinetobacter* spp., and 2 (1.3%) of 159 of nontyphoidal *Salmonella* (NTS) showed phenotypic ESBL activity.

**Conclusions:**

Our findings confirm the presence of ESBL production among pathogens causing bloodstream infections in sub-Saharan Africa. With few alternatives for managing ESBL-producing pathogens in the African setting, measures to control the development and proliferation of AMR organisms are urgently needed.

Antimicrobial resistance (AMR) is a widely acknowledged global health issue of serious concern. AMR negatively affects both individual patients and healthcare systems because community- and hospital-acquired multidrug resistant (MDR) bacterial infections are associated with increased morbidity and mortality and impose additional economic burden on healthcare systems [1–3].

Enterobacteriaceae are commonly associated with a range of infections and can rapidly develop resistance against a range of important broad-spectrum antimicrobials including extended-spectrum cephalosporins [4]. The widespread empirical use of extended-spectrum β-lactam antibiotics has inevitably led to the spread of Gram-negative bacteria expressing an array of β-lactamases, including extended-spectrum β-lactamases (ESBLs), AmpCs, and carbapenemases [5], leading to a proliferation of organisms with broad-spectrum β-lactamase activity that threatens the future of the β-Lactam class in clinical care.

Although a consensus is lacking, functional classification of ESBLs includes the β-lactamases that hydrolyze first-, second-, and third-generation cephalosporins, monobactams, and penicillins but not carbapenems [6]. However, unlike other β-lactamases, ESBLs are inhibited in vitro by β-lactamase inhibitors, such as clavulanic acid [3]. The current genotypic ESBL classification follows the Ambler classification scheme. ESBLs fall under classes A and D, whereas AmpCs fall under class C, which groups enzymes according to the molecular characterization of enzyme types [7]. The Bush/Jacoby scheme groups enzymes in phenotypic functional groups according to distinctive β-lactamase substrates [7].

There is a substantial gap in the current AMR surveillance landscape in parts of sub-Saharan Africa, and limited diagnostic options often mean that infections are managed empirically [8]. Guidelines for empiric treatment of febrile illness and septicemia such as the Integrated Management of Childhood Illness (IMCI)-based *Pocket Book of Hospital Care for Children* have not been revised in nearly 15 years, and clinicians practicing in resource-limited settings should take into consideration the most updated AMR data. Current AMR data from sub-Saharan Africa are also crucial for healthcare policy makers to prioritize resources for infection prevention and control. Utilizing data generated during the Typhoid Fever Surveillance in Africa Program (TSAP) [9], which performed standardized blood culture testing on febrile patients, we aimed to measure the burden of ESBL-producing Gram-negative bacteria associated with bloodstream infections in 12 sentinel sites in sub-Saharan Africa.

## METHODS

### Ethics

This study was approved by the Institutional Review Board (IRB) of the International Vaccine Institute as well as the IRBs and Ethics Committees of participating surveillance sites in the respective countries.

### Study Sites

Samples for this investigation were collected during the TSAP study, of which the design, methods, and site characteristics are described elsewhere [10]. Briefly, clinical and demographic data were available for 12 sentinel in- and out-patient facilities in 9 sub-Saharan African countries (Burkina Faso, Ethiopia, Ghana, Guinea-Bissau, Kenya, Madagascar, Senegal, Sudan, and Tanzania). Outpatients of all ages with a current fever at the time of consultation (tympanic or axillary temperature of ≥38.0°C or ≥37.5°C, respectively) were eligible for enrollment, as were inpatients with self-reported fever within the past 72 hours or a current fever (tympanic or axillary temperature of ≥38.0°C or ≥37.5°C, respectively). The exception was the Asante Akim North site in Ghana, which recruited children under the age of 15 years old. All participating sites followed standardized protocols and operating procedures to participate in bloodstream infection surveillance between January 2010 and September 2013. Additionally, this study included bacterial isolates from consenting patients not enrolled in the TSAP incidence analyses.

### Microbiological Procedures

Venous blood was collected from all consenting febrile patients enrolled into the study (5–10 mL for adults, 1–3 mL for children) and inoculated into an aerobic blood culture bottle for on-site incubation in a continuously monitored blood culture instrument (BD BACTEC, Becton-Dickinson, Franklin Lakes, NJ, USA, or BacT/ALERT, BioMerieux, Marcy l’Etoile, France) with the exception of Sudan, where conventional incubation and subculturing was implemented.

Blood culture bottles were incubated at 37°C for 5 days and checked daily for bacterial growth or until flagged positive in the automated system. Subculturing was done onto sheep blood agar, MacConkey agar, and chocolate agar (all Oxoid, Basingstoke, United Kingdom). Plates were incubated at 37°C for 48 hours. Bacterial isolates were identified at the sites by the analytical profile index (API 20E kits Bio-Mérieux), biochemical reactions for identification, and serogrouping if indicated. *Salmonella serovar* typing was performed at study sites using specific antisera according to standardized operating procedures.

All bacterial isolates were stored at −80°C prior to transportation to one of the reference laboratories at the Bernhard Nocht Institute for Tropical Medicine, Hamburg, Germany and the Oxford University Clinical Research Unit, Ho Chi Minh City, Vietnam, for further characterization. As described elsewhere [10], a refined list of pathogenic and likely contaminant isolates including coagulase-negative Staphylococci, *Corynebacterium* spp., *Bacillus* spp. was inferred by previous publications and consultations with experienced clinical microbiologists.

### Antimicrobial Susceptibility Testing

For pathogenic bloodstream isolates, antimicrobial susceptibility of various antimicrobial agents was reconfirmed at the reference laboratory using the modified Kirby-Bauer disk diffusion method and interpreted according to the most updated Clinical and Laboratory Standards Institute (CLSI) guidelines [11]. Phenotypic ESBL activity was suspected if ceftazidime or ceftriaxone showed resistance. Confirmation of ESBL production was performed using the double disk method where ESBL production was considered positive when a difference of ≥5 mm between the zone diameters of either the cephalosporin disks and their respective clavulanic acid combination disk was observed [11]. For the purposes of our analysis, we classified phenotypic ESBL positivity as any isolate that was resistant to either ceftriaxone or ceftazidime, and the ESBL activity was inhibited by the β-lactamase inhibitor clavulanic acid.

### PCR Screening for β-lactamase Encoding Genes

We conducted a series of multiple polymerase chain reaction (PCR) amplifications on all Gram-negative isolates to detect β-lactamase genes (CTX-M, TEM, SHV, and OXA) [12–14] and AmpC lactamase genes (MOX, DHA, EBC, FOX, ACC, CIT) [15, 16] (see Supplementary Table S1). A positive PCR result was determined by the detection of a PCR amplicon of an appropriate size for each target in the presence of appropriate positive and negative controls. Given that multiplex PCR was performed only to reveal β-lactamase groups and not the subvariant enzymes within each group, we defined genotypic ESBL positivity as any isolate that was PCR-positive for any CTX-M genes (CTX-M1, 2, 8, 9, or 25).

### Statistical Analysis

All data are presented as counts or percentages, including the proportions of specific bacteria that were reported as being susceptible to various antimicrobials (which can be seen in Table 4). Univariate analysis was performed (SAS, Cary, NC, USA version 9.4) to assess any potential associations between categorical variables and the proportion of ESBL positivity.

## RESULTS

**Figure 1.**
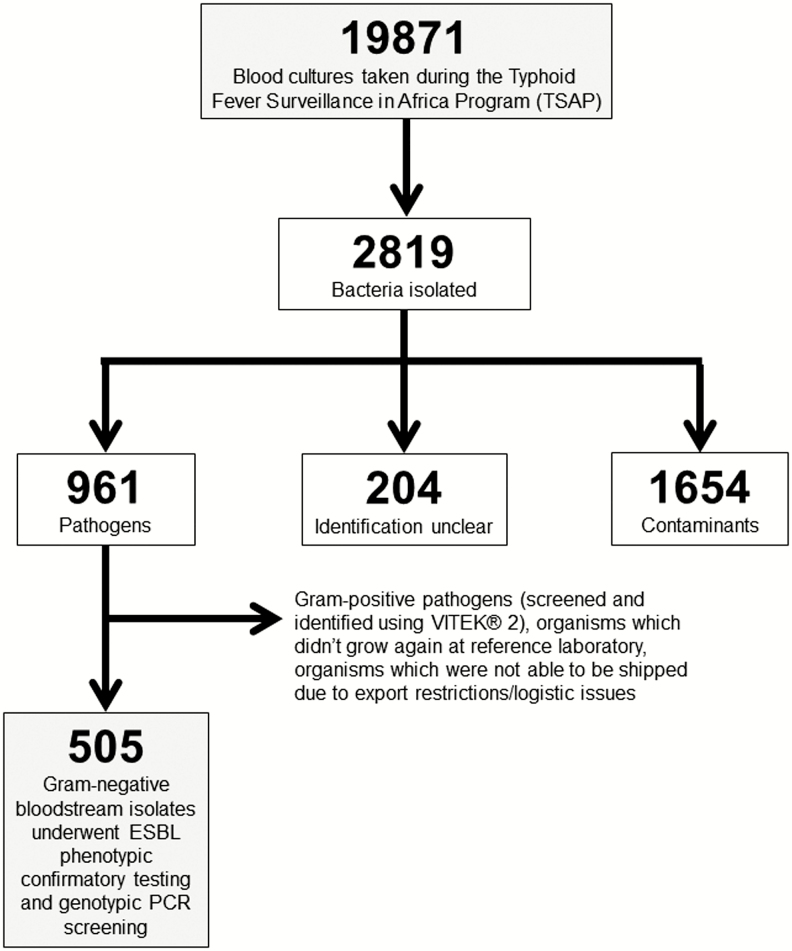
Flow diagram of bloodstream infection Gram-negative bacterial isolates for ESBL analyses. Abbreviation: ESBL, extended-spectrum β-lactamase.

Enrollment and sample collection was performed at study subjects’ first point of contact with the healthcare facility unless on an evening or weekend, in which case study staff enrolled the subject at the earliest time afterward. From 19 871 blood cultures collected during TSAP across 12 sub-Saharan Africa sites, 961 (4.8%) pathogens were isolated. For the purposes of this analysis, 505 Gram-negative bacteria were recovered, shipped, and subjected to reconfirmation at a reference laboratory—this accounted for 58.6% of all pathogens isolated during TSAP (excluding Gram-positives, unrecoverable organisms, and *Salmonella* Typhi, which were not able to be shipped due to exportation restrictions or other logistical issues) (Figure 1). The 505 pathogenic Gram-negative Enterobacteriaceae and non-Enterobacteriaceae were recovered from the blood of 396 patients in 9 African countries. Multiple pathogens (≥2 pathogenic isolates) were isolated from the blood of 83 (21.0%) of 396 patients. Enterobacteriaceae accounted for the majority 423 (84.8%) of the 505 pathogenic isolates. The majority of pathogenic isolates were from patients enrolled in Ghana, 354 (70.1%); followed by Madagascar, 39 (7.7%); Senegal, 33 (6.5%); and Burkina Faso, 27 (5.3%) of 505 (Table 1).

**Table 1.** Distribution of Various Extended-spectrum β-Lactamase Gram-negative Bloodstream Isolates and Countries of Origin

Bacteria Isolated, N	Burkina Faso	Ethiopia	Ghana	Guinea-Bissau	Kenya^a^	Madagascar	Senegal	Sudan	Tanzania	Total Isolates (%)^b^
Enterobacteriaceae										
* Enterobacter* spp.	0	0	5^c^	2	0	16	4	0	0	27 (5.3)
ESBL Phenotype pos.^d^	0	0	0	0	0	1	0	0	0	1 (3.7)
ESBL Genotype pos.^e^	0	0	2	0	0	0	2	0	0	4 (14.8)
* Escherichia coli*	7	1	85^c^	0	0	3	15	5	6	122 (24.2)
ESBL Phenotype pos.	2	0	5	0	0	0	0	0	0	7 (5.7)
ESBL Genotype pos.	1	0	4	0	0	0	0	0	0	5 (4.1)
* Klebsiella* spp.	0	0	77^c^	0	0	8	1	0	2	88 (17.4)
ESBL Phenotype pos.	0	0	39	0	0	1	0	0	0	40 (45.5)
ESBL Genotype pos.	0	0	34	0	0	2	0	0	0	36 (40.9)
iNTS	12	0	118^c^	11	1	5	8	0	4	159 (31.5)
ESBL Phenotype pos.	0	0	2	0	0	0	0	0	0	2 (1.3)
ESBL Genotype pos.	0	0	1	0	1	0	0	0	0	2 (1.3)
* Serratia* spp.	0	0	12	0	0	0	0	0	0	12 (2.4)
ESBL Phenotype pos.	0	0	2	0	0	0	0	0	0	2 (16.7)
ESBL Genotype pos.	0	0	0	0	0	0	0	0	0	0
* Proteus mirabilis*	3	0	3^c^	0	0	0	3	5	1	15 (3.0)
ESBL Phenotype pos.	1	0	0	0	0	0	0	0	1	2 (13.3)
ESBL Genotype pos.	0	0	0	0	0	0	0	0	0	0
Non-Enterobacteriaceae										
* Acinetobacter* spp.	2	0	22	7	0	3	0	3	0	37 (7.3)
ESBL Phenotype pos.	0	0	4	0	0	2	0	0	0	6 (16.2)
ESBL Genotype pos.	0	0	0	0	0	0	0	0	0	0
* Burkholderia* spp.	0	1	0	2	0	0	2	0	0	5 (1.0)
ESBL Phenotype pos.	0	0	0	0	0	0	0	0	0	0
ESBL Genotype pos.	0	0	0	0	0	0	0	0	0	0
* Pseudomonas* spp.	3	0	26	0	0	0	0	0	0	29 (5.7)
ESBL Phenotype pos.	0	0	0	0	0	0	0	0	0	0
ESBL Genotype pos.	0	0	0	0	0	0	0	0	0	0
Other^f^	0	0	6	0	0	4	0	1	0	11 (2.2)
ESBL Phenotype pos.	0	0	1	0	0	0	0	0	0	1 (9.1)
ESBL Genotype pos.	0	0	0	0	0	0	0	0	0	0
Total isolates from each country (%)	27 (5.3)	2 (0.4)	354 (70.1)	22 (4.4)	1 (0.2)	39 (7.7)	33 (6.5)	14 (2.8)	13 (2.6)	505 (100.0)
ESBL Phenotype pos. total by country (%)	3 (6.0)	0 (0)	53 (86.9)	0 (0)	0 (0)	4 (6.6)	0 (0)	0 (0)	1 (2.0)	61 (100.0)
ESBL Genotype pos. total by country (%)	1 (2.1)	0 (0)	41 (87.2)	0 (0)	1 (2.1)	2 (4.3)	2 (4.3)	0 (0)	0 (0)	47 (100.0)

Abbreviation: ESBL, extended spectrum β-lactamase; iNTS, invasive non-typhoidal *Salmonella* spp.

^a^Due to logistic constraints, ONLY *Salmonella* spp. from the Kenya site could be analyzed.

^b^Proportions for ESBL phenotype- and genotype-positives expressed as percentages of each specific species total.

^c^Includes data that was published previously by Eibach et al [20].

^d^ESBL phenotype-positive refers to resistance to ceftriaxone and susceptibility β-lactamase inhibitor (amoxicillin-clavulanate) OR resistance to ceftazidime and susceptibility to β-lactamase inhibitor (amoxicillin-clavunalate)

^e^ESBL genotype-positive refers to pathogens expressing CTX-M genes detected by polymerase chain reaction.

^f^Other non-Enterobacteriaceae includes *Achromobacter* spp., *Wautersiella* spp., *Stenotrophomonas* spp., *Pasteurella* spp., *Comamonas* spp.

Using univariate analysis, we found that organisms isolated from infants ≤1 year of age were significantly more likely to exhibit ESBL activity than those isolated from patients >1 year of age for the Ghana study site only (phenotype positive *P* = .01; and genotype positive *P* < .001, respectively) (Table 2).

**Table 2.** Age, Pretreatment, Hospital Admission, and Extended-spectrum β-Lactamase (ESBL) Status of Patients With ESBL Gram-negative Bloodstream Infections

	All Countries Except Ghana						Ghana Only					
	Phenotypic			Genotypic			Phenotypic			Genotypic		
	ESBL Positive	ESBL Negative	*P* Value	ESBL Positive	ESBL Negative	*P* Value	ESBL Positive	ESBL Negative	*P* Value	ESBL Positive	ESBL Negative	*P* Value
Age Group	n	n		n	n		n	n		n	n	
0 to ≤1 yr	3	29	.246	1	31	.667	24	76	.001	27	73	<.001
>1 yr to ≤2 yrs	1	10	.560	1	10	.457	0	27	.968	0	27	.970
>2 yrs to ≤3 yrs	0	8	.977	0	8	.979	4	17	.728	1	20	.229
>3 yrs to ≤4 yrs	0	2	.988	0	2	.989	0	4	.988	0	4	.989
>4 yrs to ≤5 yrs	0	4	.984	0	4	.985	0	3	.989	0	3	.990
>5 yrs to ≤10 yrs	2	12	.134	1	13	.620	4	9	.161	2	11	.889
>10 yrs to ≤18 yrs	0	6	.980	0	6	.981	4	11	.274	4	11	.159
>18 yrs to ≤35 yrs	1	22	.829	2	21	.303	1	20	.167	1	20	.2291
>35 yrs to ≤50 yrs	0	17	.966	0	17	.667	2	15	.599	0	17	.976
>50 yrs	0	16	.967	1	15	.457	4	38	.200	2	40	.076
Total patients (%)	7 (5.3%)	126 (94.7%)		6 (4.5%)	127 (95.5%)		43 (16.3%)	220 (83.4%)		37 (14.1%)	226 (85.9%)	
Taken antimalarial prior to consultation	1	21	.869	1	21	.993	4	24	.755	2	26	.277
Taken antimicrobial prior to consultation	2	18	.317	1	19	.909	1	22	.137	2	21	.444
Admitted to hospital	3	50	.868	3	50	.606	38	197	.820	35	200	.277

Abbreviation: ESBL, extended spectrum β-lactamase.

All organisms were screened by disk diffusion for susceptibility to ceftriaxone and ceftazidime; 136 (27%) of 505 isolates were resistant to ceftriaxone, ceftazidime, or both. Among these isolates, the activity of extended-spectrum cephalosporins was restored in vitro by clavulanic acid in 61 (44.9%) of 136 of these organisms, and these were classified to have phenotypic ESBL activity. Among the isolates resistant to a third generation cephalosporin whose activity could be restored by clavulanic acid (ESBL positive-phenotype), 23 (37.7%) of 61 isolates were also positive for a screened ESBL gene. The highest proportions of ESBL phenotype-positivity were found in *Klebsiella* spp. 40 (45.5%) of 88, *Serratia* spp. 2 (16.7%) of 12, and *Acinetobacter* spp. 6 (16.2%) of 37.

CTX-M ESBL genes were detected in 47 (9.3%) of 505 isolates. The Ghana site yielded 41 (87.2%) ESBL genotype-positive isolates, whereas Madagascar and Senegal each yielded 2 (4.3%) and Burkina Faso and Kenya each yielded 1 (2.1%) of 47 ESBL genotype-positive isolates, respectively. Among specific pathogens, ESBL genotype positivity was found in 36 (40.9%) of 88 *Klebsiella* spp., 4 (14.8%) of 27 *Enterobacter* spp., and in 5 (4.1%) of 122 *Escherichia coli*.

Multiplex PCR screening of all 505 isolates produced 189 amplicons associated with β-lactamase encoding genes (Table 3). TEM accounted for 118 (62.4%) of the 189 detected β-lactamase encoding genes and were mainly found in invasive nontyphoidal *Salmonella* (NTS) 69 (58.5%) and *E. coli* 30 (25.4%) of 118 isolates. Conversely, 12 (54.5%) of 22 OXA and 36 (76.6%) of 47 CTX-M β-lactamase encoding genes were found predominantly in *Klebsiella* spp. Overall, CTX-M β-lactamases were amplified in 47 (30.9%) of 152 Enterobacteriaceae; 45 (95.7%) of 47 belonged to the CTX-M-1 subgroup.

**Table 3.** Distribution of β-lactamase Gene Sequences Among Gram-negative Bloodstream Isolates

	Class A							Class D
Ambler Classification	TEM	SHV	CTX-M1	CTX-M2	CTX-M9	CTX-M8	CTX-M25	OXA
Enterobacteriaceae								
* Enterobacter* spp.	3	0	4	0	0	0	0	2
* Escherichia coli*	30	0	5	0	0	0	0	4
* Klebsiella* spp.	14	1	34	2	0	0	0	12
iNTS	69	1	2	0	0	0	0	4
* Serratia* spp.	0	0	0	0	0	0	0	0
* Proteus mirabilis*	1	0	0	0	0	0	0	0
Non-Enterobacteriaceae								
* Acinetobacter* spp.	0	0	0	0	0	0	0	0
* Burkholderia* spp.	0	0	0	0	0	0	0	0
* Pseudomonas* spp.	1	0	0	0	0	0	0	0
Other^a^	0	0	0	0	0	0	0	0
Total sequences	118	2	45	2	0	0	0	22

Abbreviations: CTX, cefotaxime hydrolytic activity; OXA, oxacillin hydrolytic activity; SHV, sulhydryl variable; TEM, “Temoneira” derivative.

^a^Other non-Enterobacteriaceae includes *Achromobacter* spp., *Wautersiella* spp., *Stenotrophomonas* spp., *Pasteurella* spp., *Comamonas* spp.

ESBL phenotype and genotype positivity was identified in 7 (5.7%) and 5 (4.1%) of 122 *E. coli* isolates, respectively. The susceptibility of these *E. coli* against β-lactams and β-lactamase inhibitor combinations was reduced in CTX-M PCR amplicon-positive and some amplicon-negative *E. coli* as well (Table 4). CTX-M and OXA-expressing *E. coli* were 20% and 50% susceptible to ciprofloxacin, respectively. However, the OXA-expressing organisms were susceptible to cefazolin and ceftriaxone, whereas the CTX-M expressing *E. coli* were resistant to all first-, third-, and fourth-generation cephalosporins. All β-lactamase expressing *E. coli* remained susceptible to carbapenems.

**Table 4.** Proportion of Gram-negative Bloodstream Isolates Susceptible to Antimicrobials by Ambler Classification of β-Lactamases

	Ampicillin	Amoxicillin/Clavulanate	Ampicillin/Sulbactam	Chloram-phenicol	Gentamicin	Amikacin	Ciprofloxacin	Naldixic acid	Levofloxacin	Cefazolin	Cefepime	Ceftriaxone	Ceftazidime	Ertapenem	Imipenem	Nitro-furantoin	Piperacillin/Tazobactam
Enterobacteriaceae																	
* Enterobacter* spp.																	
Class A (TEM, SHV, CTX) n = 5	0	0	n/a	20	20	100	40	27	100	0	25	20	0	100	100	50	50
CTX-M n = 4	0	0	n/a	25	25	100	50	33	100	0	33	25	0	100	100	67	67
TEM, SHV only n = 1	0	0	n/a	0	0	100	0	0	100	0	0	0	n/a	100	100	0	0
Class C (AmpC) n = 2^a^	0	0	n/a	50	100	100	50	100	n/a	n/a	n/a	50	n/a	n/a	100	n/a	n/a
Class D (OXA) n = 2^b^	0	0	n/a	0	0	100	50	0	100	0	50	0	0	100	100	100	100
Non β-Lactamase n = 22	10	17	n/a	30	29	95	60	25	95	0	58	26	12.5	100	95	21	47
* Escherichia coli*																	
Class A (TEM, SHV, CTX) n = 34	0	36	6	55	84	100	67	67	78	41	88	82	81	100	100	89	59
CTX-M n = 5	0	0	0	60	0	100	20	0	50	0	0	0	0	100	100	0	50
TEM, SHV only n = 29	0	43	6	54	94	100	75	79	81	47	100	96	96	100	100	100	60
Class C (AmpC) n = 0	…	…	…	…	…	…	…	…	…	…	…	…	…	…	…	…	…
Class D (OXA) n = 4^c^	0	0	0	25	50	100	50	25	50	100	100	100	75	100	100	100	0
Non β-Lactamase n = 86	14	46	29	49	82	93	84	72	94	66	92	87	89	98	100	83	77
* Klebsiella* spp.																	
Class A (TEM, SHV, CTX) n = 37	8	30	0	30	13	97	68	51	100	0	3	24	8	97	100	3	31
CTX-M n = 36	8	28	0	28	10	97	68	51	100	0	3	22	6	97	100	3	29
TEM, SHV only n = 1	0	100	0	100	100	100	100	100	100	0	0	100	100	100	100	0	100
Class C (AmpC) n = 1	0	0	0	0	0	100	0	0	100	0	0	0	0	100	100	0	0
Class D (OXA) n = 12^d^	17	33	0	58	18	100	33	0	100	0	0	33	0	100	100	9	45
Non β-Lactamase n = 51	12	39	19	33	41	97	61	52	90	16	35	47	36	97	100	14	49
iNTS																	
Class A (TEM, SHV, CTX) n = 70	3	59	5	13	10	9	95	90	98	5	98	92	97	100	100	18	93
CTX-M n = 2	0	0	0	0	0	0	100	100	100	0	50	50	0	100	100	50	50
TEM, SHV only n = 68	3	61	5	13	10	9	95	90	98	5	100	94	100	100	100	17	95
Class C (AmpC) n = 0	…	…	…	…	…	…	…	…	…	…	…	…	…	…	…	…	…
Class D (OXA) n = 4^e^	25	75	0	25	0	0	100	100	100	0	0	75	0	100	n/a	0	0
Non β-Lactamase n = 87	76	91	68	76	18	16	91	79	98	3	100	96	96	98	100	16	98
* Serratia* spp.																	
Class A (TEM, SHV, CTX) n = 0	…	…	…	…	…	…	…	…	…	…	…	…	…	…	…	…	…
CTX-M n = 0	…	…	…	…	…	…	…	…	…	…	…	…	…	…	…	…	…
TEM, SHV only n = 0	…	…	…	…	…	…	…	…	…	…	…	…	…	…	…	…	…
Class C (AmpC) n = 0	…	…	…	…	…	…	…	…	…	…	…	…	…	…	…	…	…
Class D (OXA) n = 0	…	…	…	…	…	…	…	…	…	…	…	…	…	…	…	…	…
Non β-Lactamase n = 12	36	91	n/a	91	100	100	64	100	100	0	100	80	100	67	100	0	100
Non-Enterobacteriaceae																	
* Pseudomonas* spp.																	
Class A (TEM, SHV, CTX) n = 1	0	0	n/a	100	n/a	n/a	100	n/a	n/a	n/a	n/a	100	n/a	n/a	n/a	n/a	n/a
CTX-M n = 0	…	…	…	…	…	…	…	…	…	…	…	…	…	…	…	…	…
TEM, SHV only n = 1	0	0	n/a	100	n/a	n/a	100	n/a	n/a	n/a	n/a	100	n/a	n/a	n/a	n/a	n/a
Class C (AmpC) n = 0	…	…	…	…	…	…	…	…	…	…	…	…	…	…	…	…	…
Class D (OXA) n = 0	…	…	…	…	…	…	…	…	…	…	…	…	…	…	…	…	…
Non β-Lactamase n = 28	10	25	35	5	100	100	95	17	86	10	85	35	83	100	100	9	70
Other^f^																	
Class A (TEM, SHV, CTX) n = 0	…	…	…	…	…	…	…	…	…	…	…	…	…	…	…	…	…
CTX-M n = 0	…	…	…	…	…	…	…	…	…	…	…	…	…	…	…	…	…
TEM, SHV only n = 0	…	…	…	…	…	…	…	…	…	…	…	…	…	…	…	…	…
Class C (AmpC) n = 0	…	…	…	…	…	…	…	…	…	…	…	…	…	…	…	…	…
Class D (OXA) n = 0	…	…	…	…	…	…	…	…	…	…	…	…	…	…	…	…	…
Non β-Lactamase n = 11	9	27	25	40	27	36	82	57	75	20	40	55	75	100	27	40	40

Abbreviations: CTX, cefotaxime hydrolytic activity; n/a, not tested; OXA, oxacillin hydrolytic activity; SHV, sulhydryl variable; TEM, “Temoneira” derivative.

^a^One AmpC strain also had CTX-M1 gene.

^b^Both OXA strains also had TEM and CTX-M1 genes.

^c^Two OXA strains also had TEM gene.

^d^All OXA had at least one other of TEM, SHV, or CTX-M genes.

^e^Two OXA strains also had TEM gene, and one of these also had CTX-M gene.

^**f**^Other non-Enterobacteriaceae includes *Achromobacter* spp., *Wautersiella* spp., *Stenotrophomonas* spp., *Pasteurella* spp., *Comamonas* spp.

When compared to *E. coli* or iNTS, the majority of *Klebsiella* spp. isolated were resistant to cephalosporins, especially OXA (33% of isolates susceptible to ceftriaxone), CTX-M (22% of isolates susceptible to ceftriaxone), and AmpC (no susceptibility to ceftriaxone)-expressing strains. The proportions of CTX-M and OXA-expressing *Klebsiella* spp. susceptible to ciprofloxacin were 68% and 33%, respectively.

Combined ESBL phenotype and genotype positivity was found in 2 (1.3%) of 159 iNTS isolates. Serotyping was only performed on 63 of the nontyphoidal *Salmonella* isolates. This led to 34 *Salmonella serovar* Typhimurium, 15 *Salmonella serovar* Dublin, 9 *Salmonella serovar* Enteritidis, and 5 other unknown serotypes. Among serotyped iNTS, one *Salmonella serovar* Typhimurium from Kenya was ESBL genotype-positive; the other iNTS ESBL isolates were not serotyped. None of the CTX-M iNTS isolates were susceptible to amoxicillin/clavulanate (Table 4), and iNTS susceptibility to fluoroquinolones was 95% and above for all isolated strains (including CTX-M strains). All iNTS phenotypes were susceptible to carbapenems.

## DISCUSSION

The sub-Saharan Africa genotypic ESBL prevalence of 9.3% and phenotypic ESBL prevalence of 12.1% from our findings appear to be within the lower range of other available estimates [17–19]. It should be of note that 354 (70.1%) of 505 total bacteria isolated were identified through the Asante Akim North, Ghana surveillance site. Of the Ghanaian bloodstream Enterobacteriaceae isolates, 48 (16.0%) of 300 were ESBL phenotype-positive including two iNTS ESBL phenotype-positive isolates, slightly higher than the 41 (9.7%) of 423 described by Eibach et al (2016) in previous surveillance in a similar region in Ghana [20]. This may reflect differences in our “Enterobacteriaceae pathogen mix” gathered from Ghana—the total number of samples gathered by Eibach and colleagues contained a much larger proportion of ESBL-negative *Salmonella* enterica. That analysis included 215 iNTS isolates and 110 *Salmonella* Typhi isolates, whereas our analysis only included 159 iNTS isolates. This may be due to the facts that Eibach and colleagues additionally included isolates from 2007 to 2009 and logistical issues in our analysis, which did not make it possible to include any *Salmonella serovar* Typhi isolates here.

The majority of iNTS, *Klebsiella* spp., and *E. coli* isolates in our analysis carried TEM, CTX-M-1, and/or OXA β-lactamase genes. iNTS (69, 90.8% of 76) and *E. coli* (30, 76.9% of 39) isolates in general were found to have more TEM-type genes, whereas *Klebsiella* spp. isolates carried more OXA and CTX-M-type genes (Table 2). This is reflected phenotypically as lower proportions of *Klebsiella* spp. were found to be susceptible to ceftriaxone and ceftazidime compared to other Enterobacteriaceae (Table 4). Among the CTX-M group, the predominance of the CTX-M-1 subgroup could suggest the presence of CTX-M-15 enzyme, which is the most widely disseminated CTX-M ESBL enzyme [21].

Reduced susceptibility to β-lactams was often coupled with reduced fluoroquinolone susceptibility for *E. coli* and *Klebsiella* spp. isolates (Table 4). Although some clinicians may consider a fluoroquinolone before a carbapenem for empiric treatment of ESBL bacteremia because of availability and cost, this may be associated with an increased mortality even in strains where fluoroquinolones are reported as susceptible [22]. Unfortunately, this would leave parenteral carbapenems as one of the last viable treatment options for many affected patients like those identified in our surveillance.

As expected, all pathogens isolated with CTX-M genes were resistant to early generation cephalosporins and the majority of these were also resistant to extended-spectrum cephalosporins. This finding was expected as it is known that CTX-M β-lactamases have greater activity in hydrolyzing third- and fourth-generation cephalosporins. In contrast, TEM and SHV isolates (iNTS and *E. coli*), which are known to hydrolyze penicillins and lower generation cephalosporins, retained susceptibility to extended-spectrum cephalosporins. Traditionally, OXA β-lactamases do not hydrolyze extended-spectrum cephalosporins well, so our findings of resistance against antimicrobials such as cefepime and ceftazidime, particularly among iNTS, *Enterobacter*, and *Klebsiella* isolates, are cause for concern. Interestingly, OXA-expressing *E. coli* isolates retained susceptibility to extended-spectrum cephalosporins. This complicates clinicians turning to carbapenems (which is unlikely to be a practical choice in many underresourced settings) as one of the last line options for treatment against MDR Gram-negatives unless there are either methods available for phenotypic or genotypic ESBL identification or clinical deterioration despite therapy with extended-spectrum cephalosporins. Our data indicate that the proportion of ESBL (and OXA β-lactamase) positive *E. coli* and iNTS is still low; this, taken together with the remainder of our Gram-negative ESBL data, does not yet support empiric treatment with carbapenem agents.

ESBL positivity and age-association were analyzed separately for Ghana and non-Ghana sites because only children under 15 years old were recruited in the Asante Akim North site. The reason for the association between age up to 1 year old and ESBL positivity in the Ghana site is unclear—to our knowledge there has been no association found to date linking infants of young age to ESBL positivity. Many clinical and demographic data that would have shed light on this association were either missing or not collected as a part of surveillance. It is also possible that associations between other age groups and ESBL positivity exist, but our study was not powered to detect them.

Our findings reveal further data on the distribution of AMR in sub-Saharan Africa. Previous data from Africa suggest that the proportion of ESBL producing bloodstream isolates can be quite variable; perhaps the most comprehensive review of ESBLs in Africa (13 countries) was performed by Tansarli et al in 2013, which reported 0.7–75.8% of Enterobacteriaceae to be ESBL producing isolates in bloodstream infections [19]. However, none of these sources reported in detail both genotypic and phenotypic data for ESBL isolates. In general, our findings indicate that >1 out of 10 (12.1%) of Gram-negative pathogens are ESBL phenotype positive. Moreover, because blood samples were collected at the subjects’ first point of contact with the healthcare facility, it can be inferred that all isolates were community-acquired. ESBL phenotype positivity was not limited to any specific pathogen within the Enterobacteriaceae family and also included non-Enterobacteriaceae such as *Acinetobacter*. Although these pathogens may have benefitted from prompt treatment with carbapenems, capacities of microbiology laboratories should be first prepared to perform phenotypic ESBL screening and confirmatory testing as a tool to promote antimicrobial stewardship and prevent untoward empiric use of carbapenems, which are expensive and broad-spectrum in their activity.

Our study has several limitations. First, in certain surveillance settings, there were some patients included in this analysis who were enrolled without meeting the TSAP inclusion criteria, which may have compromised the homogeneity of sample collection and the generalizability of results. This was rationalized because it was not ethically acceptable to withhold access to blood culture for patients who would benefit from the test. Second, we were not able to report the genotypic subvariants within TEM, CTX-M, SHV, and OXA groups at the time of writing. Therefore, we counted all isolates with non-CTX-M gene groups as ESBL genotype-negative, and it is possible that this could have underestimated true ESBL genotype-positivity. Genome sequencing work is being performed on isolates from surveillance that will reveal genotypic variants. Third, logistic challenges limited our analyses to the pathogens that were able to be received at the reference laboratory. Although such data would have been of importance, we were unfortunately not able to perform any ESBL analyses on *Salmonella* Typhi or Paratyphi isolates due to these challenges. For this reason, the 505 isolates included in these analyses may not necessarily be representative of the pathogen mix or distribution of pathogens for each country.

In summary, our findings are broadly consistent with the sparse ESBL data available and reveal ESBL production in a substantial minority of pathogenic Gram-negative bloodstream isolates collected from sentinel surveillance sites in sub-Saharan Africa. Resistance to ESBL agents may have particular relevance for guidelines such as IMCI, which outline the empiric management of febrile illnesses and suspected septicemia. Our data suggest that in >1 in 10 Gram-negative bloodstream isolates, Carbapenems may be one of the last susceptible alternatives even though these agents are expensive, not readily available, and must be administered parenterally. The prevalence of ESBL bloodstream pathogens requiring carbapenems as first-line agents in resource-limited settings should be a great cause for concern, not only for the factors just mentioned but also because it may lead to the more serious problem of carbapenemase-producing *Enterobacteriaceae*. To this end, integrated antimicrobial stewardship measures become even more paramount to ameliorate the burden of AMR. Although much work needs to be done to develop new classes of antimicrobials, control strategies must also be integrated with improved water and sanitation as well as vaccines (such as newly available typhoid conjugate vaccines) where applicable.

## Supplementary Data

Supplementary materials are available at *Clinical Infectious Diseases* online. Consisting of data provided by the authors to benefit the reader, the posted materials are not copyedited and are the sole responsibility of the authors, so questions or comments should be addressed to the corresponding author.

ciz450_suppl_Supplemental_Table_S1

## Notes

***Financial support.*** This research was funded by the Bill & Melinda Gates Foundation (OPP1127988). The International Vaccine Institute acknowledges its donors including the Republic of Korea and the Swedish International Development Cooperation Agency. This publication was made possible through a grant from the Bill & Melinda Gates Foundation (OPP1201031).

***Supplement sponsorship.*** This article was published as part of the supplement “Severe Typhoid Fever in Africa (SETA) Program” sponsored by the International Vaccine Institute.

***Potential conflicts of interest.*** All authors: No potential conflicts of interest. All authors have submitted the ICMJE Form for Disclosure of Potential Conflicts of Interest. Conflicts that the editors consider relevant to the content of the manuscript have been disclosed.
