# Supplementary material for: Multicountry Distribution and Characterization of Extended-spectrum β-Lactamase–associated Gram-negative Bacteria From Bloodstream Infections in Sub-Saharan Africa
Source: Clin Infect Dis. 2019 Oct 30;69(Suppl 6):S449–58. doi: 10.1093/cid/ciz450 (PMC6821266; doi:10.1093/cid/ciz450)
Supplement: ciz450_suppl_Supplemental_Table_S1 [file ciz450_suppl_supplemental_table_s1.docx]

**Table S1. Primer Sequencing**

|  | **Primers** | **Sequence (5' - 3')** | **Size (bp)** | **Notes** | **Ref.** |  |
| --- | --- | --- | --- | --- | --- | --- |
| 1 | CTX-M-1F | AAAAATCACTGCGCCAGTTC | 415 | Multiplex PCR  for CTX-M | Woodford *et al.* 2005 | |
|  | CTX-M-1R | AGCTTATTCATCGCCACGTT |  |  |  |  |
| 2 | CTX-M-2F | CGACGCTACCCCTGCTATT | 552 |  |  |  |
|  | CTX-M-2R | CCAGCGTCAGATTTTTCAGG |  |  |  |  |
| 3 | CTX-M-9F | CAAAGAGAGTGCAACGGATG | 205 |  |  |  |
|  | CTX-M-9R | ATTGGAAAGCGTTCATCACC |  |  |  |  |
| 4 | CTX-M-8F | TCGCGTTAAGCGGATGATGC | 666 |  |  |  |
| 5 | CTX-M-25F | GCACGATGACATTCGGG | 327 |  |  |  |
|  | CTX-M-8/25R | AACCCACGATGTGGGTAGC |  |  |  |  |
| 6 | MOXF | GCTGCTCAAGGAGCACAGGAT | 520 | Multiplex PCR  for AmpC | Perez-Perez *et al.* 2002 | |
|  | MOXR | CACATTGACATAGGTGTGGTGC |  |  |  |  |
| 7 | CITF | TGGCCAGAACTGACAGGCAAA | 462 |  |  |  |
|  | CITR | TTTCTCCTGAACGTGGCTGGC |  |  |  |  |
| 8 | DHAF | AACTTTCACAGGTGTGCTGGGT | 405 |  |  |  |
|  | DHAR | CCGTACGCATACTGGCTTTGC |  |  |  |  |
| 9 | ACCF | AACAGCCTCAGCAGCCGGTTA | 346 |  |  |  |
|  | ACCR | TTCGCCGCAATCATCCCTAGC |  |  |  |  |
| 10 | EBCF | TCGGTAAAGCCGATGTTGCGG | 302 |  |  |  |
|  | EBCR | CTTCCACTGCGGCTGCCAGTT |  |  |  |  |
| 11 | FOXF | AACATGGGGTATCAGGGAGATG | 190 |  |  |  |
|  | FOXR | CAAAGCGCGTAACCGGATTGG |  |  |  |  |
|  | TEMF | CATTTCCGTGTCGCCCTTATTC | 800 | Multiplex PCR  for TEM, SHV,  OXA | Dallenne *et al.* 2010 | |
|  | TEMR | CGTTCATCCATAGTTGCCTGAC |  |  |  |  |
|  | SHVF | AGCCGCTTGAGCAAATTAAAC | 713 |  |  |  |
|  | SHVR | ATCCCGCAGATAAATCACCAC |  |  |  |  |
|  | OXAF | GGCACCAGATTCAACTTTCAAG | 564 |  |  |  |
|  | OXAR | GACCCCAAGTTTCCTGTAAGTG |  |  |  |  |
